# Cancer Therapy-Induced Encephalitis

**DOI:** 10.3390/cancers16213571

**Published:** 2024-10-23

**Authors:** Nicolas P. Desbaillets, Andreas F. Hottinger

**Affiliations:** 1 Department of Oncology, Centre Hospitalier Universitaire Vaudois (CHUV), Lausanne University, 1011 Lausanne, Switzerland; 2 Lundin Family Brain Tumor Research Centre, Centre Hospitalier Universitaire Vaudois (CHUV), Lausanne University, 1011 Lausanne, Switzerland; 3 Department of Clinical Neurosciences, Centre Hospitalier Universitaire Vaudois (CHUV), Lausanne University, 1011 Lausanne, Switzerland

**Keywords:** cancer, adverse event, neurotoxicity, encephalitis, autoimmune, immunotherapy, checkpoint inhibitor, CAR-T, ICANS, BiTE

## Abstract

Encephalitis associated with cancer therapies is a rare but serious complication that can significantly impact patients’ quality of life and it requires prompt identification and management. Over the past two decades, immunotherapy—particularly immune checkpoint inhibitors—has become a cornerstone of cancer treatment, with up to half of metastatic cancer patients in economically developed countries now receiving these therapies. The widespread adoption of immunotherapy has led to improved survival rates and long-term remissions, even in patients with advanced metastatic disease. However, as immune modulators, these therapies can trigger a range of immune-related adverse events, including a variety of novel neurological toxicities. Among these, encephalitis is of particular concern due to its potential severity, which can compromise treatment outcomes. This review aims to provide a comprehensive overview of the literature on this condition, highlighting optimal diagnostic strategies and management approaches to mitigate the risk of significant morbidity, while also comparing encephalitis induced by immunotherapy with that caused by traditional chemotherapies and targeted oncologic treatments.

## Simple Summary

Cancer immunotherapies, while revolutionary in boosting the immune system to fight cancer, can also trigger serious side effects, including inflammation of the brain. We review the existing literature on encephalitis with a special focus on cancer treatment-related encephalitis, including its etiopathogenesis, differential diagnosis, and identification of optimal diagnostic approaches and treatment strategies. This review aims to help clinicians from oncology and neurology improve the diagnosis and management of this condition, help reduce complications, improve patient outcomes, and inform future therapeutic approaches.

## 1. Introduction

### 1.1. Encephalitis

Encephalitis is an inflammation of the brain parenchyma, often involving the cortical or deep gray matter, with or without the involvement of white matter, meninges, or spinal cord. It generally results from a direct viral infection or an autoimmune process that may be postinfectious, paraneoplastic, or idiopathic. The typical clinical presentation is a combination of fever, headache, and altered mental status (behavior, personality, cognition, and consciousness). Focal neurological deficits, seizures, movement disorders, and/or autonomic instability are often associated. Unfortunately, symptoms remain largely nonspecific, and given the multiple etiologies, diagnosis of encephalitis remains challenging [1]. The typical diagnosis relies mostly on clinical symptoms and workup requires serum and cerebrospinal fluid (CSF) analysis, neuroimaging, and electroencephalography (EEG) [2]. Autoantibody testing should be considered in all cases, especially those with a recognizable autoimmune encephalitis phenotype [3].

Based on the International Encephalitis Consortium [4], encephalitis is defined as an altered mental status lasting 24 h or more, without alternative cause and at least two of the following:

- Fever above 38 °C within the last 72 h.
- New focal neurological signs.
- New epileptic seizure activity, unrelated to a pre-existing condition
- Signs of encephalitis on MRI.
- Abnormal EEG findings consistent with encephalitis.
- CSF pleocytosis.

However, most patients with encephalitis will not present a severely depressed Glasgow coma scale (GCS) score on admission and may even perform well on basic cognitive assessments such as mini-mental-status (MMS), or Montreal Cognitive Assessment (MoCA) tests, and might, especially in autoimmune or paraneoplastic forms, lack fever or CSF pleocytosis [1].

Encephalitis can be broadly classified as infectious or autoimmune [5,6]. Infectious encephalitis is beyond the scope of this review, is mostly viral in origin, and has been reviewed by Tyler et al. in the *New England Journal of Medicine* [2]. Autoimmune encephalitis (AE) is a group of non-infectious immune-mediated inflammatory diseases. In some (but not all) cases of AE, a specific autoantibody directed against a CNS antigen can be detected in the serum or cerebrospinal fluid [7]. The most common etiologies are associated with antibodies against leucine-rich glioma-inactivated 1 (LGI1) and contactin-associated protein-like 2 (CASPR2), and in patients under 50, autoantibodies against the N-methyl-D-aspartate receptor (NMDAR) are observed at similar frequencies [8]. In addition to these two autoantibodies, more than a dozen new types of autoantibodies have been identified in the last 15 years, and their incidence is increasing [7]. These antibodies are typically directed against extracellular domains of neuroglial proteins [9].

Autoimmune encephalitis is often observed as a paraneoplastic syndrome. One of the best and most comprehensively described models for this is the association of ovarian teratomas with anti-NMDAR AE [10], with up to 25% of patients with anti-NMDAR AE having an ovarian teratoma [11]. Paraneoplastic neurological disorders are usually subacute at onset, progressive due to accelerated neuronal death, have a poor prognosis, and are typically detected months to years before a tumor is diagnosed [12,13]. Brain imaging may show no abnormal findings. Testing for anti-NMDAR antibodies in the cerebrospinal fluid may be useful, but is not necessary to make a diagnosis of CNS toxicity related to an autoimmune process [14]. In contrast to classical autoimmune encephalitis, patients with paraneoplastic etiology show significantly less clinical improvement when treated with steroids and intravenous immunoglobulin [15]. In paraneoplastic encephalitis, a cancer elicits an immune-mediated response directed against CNS antigens, primarily by molecular mimicry. Specific autoantibodies associated with particular tumors (Table 1) are typically directed against intracellular targets [13]. For example, up to 50% of patients with gamma-aminobutyric acid B receptor [GABABR] [16] and α-amino-3-hydroxy-5-methyl-4-isoxazolepropionic acid receptor (AMPAR) [17] encephalitis have been shown to have cancer. Notably, the presence of onconeural antibodies such as anti-Hu and anti-Ma2 are almost always associated with an underlying cancer in adults [13]. Interestingly, the incidence of such paraneoplastic encephalitis increases with checkpoint inhibitors [18,19] and is discussed below.

In addition to the encephalitis described above, which is better defined as limbic encephalitis, paraneoplastic neurologic syndromes may include encephalomyelitis, sensory neuronopathy, opsoclonus-myoclonus, enteric neuropathy, rapidly progressive cerebellar syndrome, and Lambert–Eaton myasthenic syndrome. These conditions have been reviewed by Graus et al. [13]. The term encephalomyelitis (EM) only applies to patients presenting neurological dysfunction at multiple sites, affecting both central and peripheral nervous systems, including dorsal root ganglia, peripheral nerve, or nerve roots [13]. Rapidly progressive cerebellar syndrome (RPNS), as its name suggests, is a rapidly progressive, severe, and bilateral cerebellar syndrome limiting daily activities in less than 3 months and is characterized by the absence of substantial cerebellar atrophy on imaging [13]. RPNS has only been described in small patient series but appears to be associated with anti-Yo and Tr/delta/notch-like epidermal growth factor-related receptor (DNER) antibodies [13]. Opsoclonus-myoclonus syndrome (OMS) consists of involuntary, chaotic, multidirectional, high-frequency, saccadic movements of the head, trunk, and limbs. Signs of encephalopathy (ranging from confusion to coma) and cerebellar involvement (dysarthria or truncal ataxia) have also been reported. Paraneoplastic OMS in adults is most often associated with SCLC or breast cancer and is mostly associated with anti-Ri antibodies [13].

Finally, acute demyelinating encephalomyelitis (ADEM) also constitutes an important cause of non-infectious encephalitis. ADEM is defined as an autoimmune demyelinating disease caused by an autoimmune response. This immune response is thought to be post-infectious or following vaccination, although this has never been proven. It presents as an acute onset encephalopathy associated with polyfocal neurologic deficits preceded by fever, malaise, irritability, somnolence, headache, nausea, and vomiting. It is seen primarily in pediatric populations and is often associated with antibodies to myelin oligodendrocyte glycoprotein [20].

### 1.2. Immune Checkpoint Inhibitors

Over the past two decades, immune checkpoint inhibitors (ICIs) have become a mainstay of cancer therapy, with up to half of metastatic patients in economically developed countries now receiving ICIs [21]. ICIs enhance antitumor immunity by blocking immune checkpoint molecules expressed on the surface of T lymphocytes and tumor cells, such as cytotoxic T lymphocyte-associated antigen 4 (CTLA-4), programmed cell death protein 1 (PD-1), and its ligand (PD-L1) [22]. Today, more than 50 different malignancies can be treated with 10 approved ICIs either as monotherapy or in combination in the neoadjuvant, adjuvant, or palliative setting [23,24] (Table 2). The introduction of ICIs into routine oncology practice has led to increased survival and long-term remissions, even in patients with extensive metastatic cancer [21].

ICIs are surface receptors expressed by immune cells that regulate immune homeostasis. PD-1 and its primary ligand PD-L1 are expressed on T cells and tumor cells, respectively, resulting in T cell exhaustion upon interaction. This mechanism is exploited by tumor cells to avoid immune elimination [21]. Thus, PD-1/PD-L1 like LAG-3 inhibition acts directly at the tumor site, whereas CTLA-4 inhibition acts mainly during T cell priming when antigen-presenting cells (APC) and the T cells interact in lymph nodes.

## 2. Checkpoint Inhibitor-Induced Encephalitis

Although very beneficial, ICIs come at a cost: A major drawback is the development of immune-related adverse events (irAEs), including serious (grade 3 or higher) neurologic syndromes (1–3% of cases) [25,26,27,28]. Although neurologic adverse events from ICIs are less common than those from chemotherapy, they must be considered, as they account for 11% of all fatal adverse events linked to ICIs. [24]. These include the exacerbation of pre-existing and the de novo development of autoimmune neurological diseases of a central and/or peripheral nature.

Typically, ICIs have been associated with peripheral neuropathies, Guillain-Barré syndrome, myasthenia gravis, Tolosa–Hunt syndrome, and autoimmune encephalitis [25,27]. Checkpoint inhibitor-induced encephalitis (CIIE) is a rare (0.1–1%) but serious complication of checkpoint inhibitor immunotherapy, with increasing incidence with concurrent or sequential ICI treatment [29]. Interestingly, although the majority of autoimmune diseases are diagnosed in women, gender has not been found to be a specific risk factor for immune-related adverse events linked to checkpoint inhibitors [30]. With regard to encephalitis specifically, sufficient demographic data are lacking. Moreover, ICI is mostly used in lung, melanoma, and urinary tract tumors that typically predominantly affect men, the gender-specific analysis of adverse events is inherently biased. Removal of such a confounding factor will require large patient numbers that will only be obtained once very large data series of those rare side effects linked to ICIs are available.

The exact mechanism by which checkpoint inhibitors lead to CIIE is not fully understood. It is thought to involve an immune response to brain tissue, as seen in autoimmune and paraneoplastic encephalitis. Risk factors for developing CIIE include a history of autoimmune disease, prior radiation therapy, and concurrent infections.

As an illustrative case, a patient with pulmonary adenocarcinoma and type 1 diabetes developed GAD65 antibody-positive encephalitis while receiving nivolumab [31]. GAD65 is expressed on pancreatic islet cells and GABAergic neurons. In seropositive cases, autoantibodies against neuronal surface/synaptic antigens in the limbic system are usually found [32,33]. Mechanistically, antibody/antigen binding can lead to complement deposition, antigen internalization, and modulation of the function of the antigenic target. The origins and sources of these autoantibodies are generally unclear but may be secondary to previous infections, or paraneoplastic [34]. In contrast to classic PNS, which are known to precede the detection of cancer, ICI-induced neurological syndromes, by definition, develop when the cancer is already diagnosed, generally within weeks to months after the initiation of ICIs. Whether pre-existing autoreactivity is unleashed or, on the contrary, generated de novo remains unclear.

Recent data showed an increased incidence of anti-Ma2 autoimmune limbic encephalitis in patients receiving nivolumab, pembrolizumab, or a combination of ipilimumab and nivolumab [35]. A case of anti-NMDAR positive CSF in a melanoma patient treated with checkpoint inhibitors has been reported by Williams et al. [14]. We have published a case of anti-Hu positive limbic encephalitis in a patient with NSCLC who was treated with ipilimumab and nivolumab [36]. However, many cases remain seronegative despite thorough screening and antibody detection is not required to classify symptoms as irAEs. In a review of 47 cases of CIIE by Stuby et al., one or more autoantibodies (anti-CASPR2, anti-GAD65, anti-AGNA, anti-Hu, anti-NMDAR, anti-Ma2, anti-Ri, and anti-TPO/TG) were detected in 52% of patients treated with ipilimumab, nivolumab, pembrolizumab, or atezolizumab. Moreover, 29/47 cases (62%) were identified in anti-PD-1 ICI monotherapy patients and 5/47 cases (11%) in anti-CTLA-4 ICI monotherapy patients. The concurrent or sequential use of CTLA-4 and PD-1 inhibitors caused encephalitis in 9/47 cases (19%) [37]. In a more recent series by Farina et al., with 71 cases of CIIE mostly with single agent anti-PD1/PD-L1 ICI, anti-paraneoplastic autoantibodies (anti-Hu, Ma2, and Yo antibodies) were found in 84% of patients with focal encephalitis. In meningoencephalitis patients, however, 37% remain antibody-negative, 25% with non-specific brain reactive autoantibodies, and 33% with non-paraneoplastic autoantibodies (such as anti-GAD65) [38].

There is growing interest in the relationship between the gut–brain axis, neuroinflammation, and the role of ICIs. The microbiota–gut–brain axis is a complex and multi-faceted network that connects the brain and gut through a variety of immunological, hormonal, and neural signals. An imbalance in gut microbiota (dysbiosis) can affect and trigger neuroinflammation and microglial activation [39]. In murine models, alteration in the gut–brain axis has been shown to disrupt the blood–brain barrier and to be linked to CNS autoimmune diseases such as encephalomyelitis and multiple sclerosis [40]. Similarly, it is postulated that the gut microbiota may influence the effectiveness of ICIs by directly regulating immune cells and modulating the immune system through the gut–brain axis [41]. Whether the gut–brain axis and the microbiota play a role in CIIE remains largely unknown but represents an important emerging topic of research.

### 2.1. Diagnosis of CIIE

Diagnosis of CIIE can be challenging because symptoms can be subtle and indistinguishable from other causes of encephalitis, including viral infections. Any patient with altered mental status and fever without an obvious cause should be treated as having a central nervous system (CNS) infection until proven otherwise [42]. CIIE remains a diagnosis of exclusion (Table 3), but it is important for healthcare providers to consider CIIE in the differential diagnosis of patients receiving checkpoint inhibitors who present with a decreased level of consciousness, confusion, headache, fever, seizures, and/or focal neurologic deficits. The time interval between initiation of ICI therapy and the onset of symptoms is highly variable, ranging from less than a week to up to 2 years, with a median of 6 to 9 weeks [27,37]. In cases of uncertainty, neuronal damage biomarkers such as neurofilament light chain (NfL) and S100B can quickly signal neuroaxonal damage. These markers, though non-specific, help in flagging patients for further neurological evaluation, particularly when antibody tests are negative [43].

Diagnosis is usually based on a combination of clinical presentation, imaging studies, and laboratory tests. CSF analysis must be performed to rule out infectious etiologies (bacterial, fungal, or viral meningoencephalitis) and leptomeningeal disease. Typical CSF findings in patients with CIIE are pleocytosis with predominantly lymphocytes and elevated protein concentrations. Glucose levels are unaffected. CSF polymerase chain reaction (PCR) for herpes simplex virus type 1 (HSV-1), herpes simplex virus type 2 (HSV-2), and varicella-zoster virus (VZV) should be ordered. Since we are dealing with cancer patients, a cytological examination of the cerebrospinal fluid is recommended.

**Figure 1:**
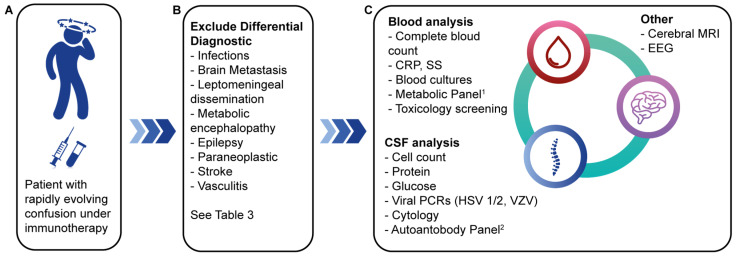
Diagnosis of ICI-induced encephalitis. (**A**) Early symptoms, especially confusion, must raise suspicion of encephalitis. (**B**) Differential diagnosis to be evoked; (**C**) workup to be done. ^1^ Metabolic panel: electrolytes, glucose, creatinine, bilirubin, liver enzymes, ammonia, arterial blood gas analysis, vitamins B1 and B12, thyroid function tests, basal cortisol concentration, ANCA; ^2^ autoantibody panel: autoantibodies: AGNA (SOX1), AMPAR1/2, Amphiphysin, CRMP5 (CV2), DPPX, GABAR, GAD65, Hu (ANNA1), Ma1, Ma2, Ri (ANNA2), TG, TPO, TR, VGKC (anti-LGI1, anti-CASPR2), Yo (PCA1), ZIC44; Paraneoplastic antibodies: NMDAR, AMPAR1/2, VGKC (anti-LGI1, anti-CASPR2), Gly, GABAR. Abbreviations: ANCA = antineutrophil cytoplasmic antibody; CRP = C-reactive protein; CSF = cerebrospinal fluid, EEG = electroencephalogram > HSV-1 = herpes simplex virus type 1; HSV-2 = herpes simplex virus type 2, MRI = magnetic resonance imaging; PCR = polymerase chain reaction; SS = sedimentation speed; VZV = varicella-zoster virus. Adapted from [37].

A cerebral MRI is part of the workup to rule out brain metastases, ischemia, and intracranial hemorrhage. An EEG can rule out nonconvulsive status epilepticus. A complete laboratory work-up is performed, including a complete blood count, a C-reactive protein (CRP) test, a comprehensive metabolic panel (including ammonia), blood concentrations of vitamins (B1, B12), an evaluation of the pituitary hormonal axes with thyroid-stimulating hormone (TSH) and cortisol. Blood and urine cultures should be obtained. Antineutrophil cytoplasmic antibody (ANCA) titers should be measured to rule out vasculitis. If intoxication is suspected, a toxicology screen should be performed [28,44]. There are no specific tests for CIIE; in the case series by Stuby et al., less than 50% of performed MRIs revealed findings consistent with encephalitis. No encephalitis-specific EEG pattern was found, although epileptiform spikes were seen in 30% of EEGs. CSF pleocytosis and elevated CSF protein levels were found in a majority of patients [37]. A summary of the diagnostic approach and workup is illustrated in Figure 1.

### 2.2. Management of CIIE

Immune-mediated encephalitis is generally graded from 3 to 4 and requires permanent discontinuation of ICI. The standard of care is corticosteroids, which should be started as soon as possible after differential diagnoses have been ruled out. The search for autoimmune encephalitis or paraneoplastic antibodies should not delay the initiation of treatment, as they can be quantified even in the presence or after corticosteroids. Intravenous methylprednisolone at a dose of 1 to 2 mg/kg body weight/day is recommended initially [45,46]. In severe cases, the corticosteroid dose may be increased to ≥10 mg/kg daily. With such doses of corticosteroids, adverse events such as delirium, insomnia, hyperglycemia, or immunosuppression should be expected. As usual, in immunosuppressed patients, prophylaxis with trimethoprim/sulfamethoxazole (300 mg, three times a week) and acyclovir (500 mg daily) is indicated. In some cases, corticosteroid treatment becomes chronic, but ideally, treatment should be discontinued if possible. In this case, the steroids should be slowly tapered over a period of at least four weeks [45,46].

In steroid-refractory cases, treatment with intravenous immunoglobulins (IVIG), plasmapheresis, or rituximab should be considered [1,45,47]. We have successfully treated an NSCLC patient with anti-Hu-positive autoimmune limbic encephalitis on ipilimumab and nivolumab with natalizumab, an antibody that blocks the α4β1 integrin on blood–brain barrier endothelial cells, limiting lymphocyte recruitment into the CNS [36].

It is important for healthcare providers to be aware of the risk of CIIE and to closely monitor patients receiving checkpoint inhibitor immunotherapy for signs or symptoms of this complication. Early detection and treatment of CIIE can help improve patient outcomes. Early diagnosis and appropriate treatment improve patient outcomes, and symptom control is achieved in the majority of patients (82%). However, despite optimal management and supportive care, CIIE remains a serious complication with an in-hospital mortality of nearly 20%, depending on the series [37].

### 2.3. Monitoring of the Evolution of CIIE

Neural antibodies are well-recognized biomarkers of autoimmune and paraneoplastic encephalitis and treatment response and outcome largely depend on the targeted antigen. Antibodies against intracellular antigens (anti-Hu, Ma2, Yo) are associated with poor outcomes whereas antibodies against cell-surface antigens (anti-NMDAR, LGI1, CASPR2) are associated with more favorable outcomes [43]. Likewise, the prognostic value of neural antibodies has also been demonstrated in CIIE. Paraneoplastic antibodies were found associated with a lower probability of treatment response and higher risk of mortality as opposed to GFAP or neuron-surface antibodies [38].

## 3. Immunotherapy-Induced Acute Demyelinating Encephalomyelitis

Although atypical, Zafar et al. described a case of nivolumab-induced acute demyelinating encephalomyelitis (ADEM) [15]. Two weeks after starting palliative nivolumab immunotherapy, a patient with relapsed and metastatic laryngeal squamous cell carcinoma exhibited progressive weakness, altered mental status, and progressive dyspnea requiring intubation. A cMRI showed multiple hyperintense T2 flair white matter lesions without contrast enhancement or restricted diffusion, consistent with acute demyelinating encephalomyelitis. Similar to the encephalitis cases above, 5 days of intravenous methylprednisolone and immunoglobulin (IVIg) therapy allowed gradual improvement of motor symptoms.

## 4. Activated T Cell-Induced Encephalitis

### 4.1. Chimeric Antigen Receptor T-Cells

Chimeric antigen receptor T-cells (CAR-T cells) are a new approach in clinical cancer therapy and a form of tumor immunotherapy. The basic idea behind this approach is to harvest the patient’s T cells, provide them with receptors specific for tumor surface markers, and return them to the patient where they will eliminate tumor cells.

CAR-T cells are genetically engineered to express a chimeric transmembrane receptor that includes an extracellular single-chain variable fragment (scFv) targeting tumor antigens. This scFv fragment is linked via a hinge and transmembrane domain to intracellular T cell signaling domains. These signaling domains comprise the CD3 zeta chain (CD3ξ) of the T-cell receptor (TCR), which contains the immunoreceptor tyrosine-based activation motif (ITAM), as well as costimulatory domains from CD28 and 4-1BB. To date, all approved CAR-T cell therapies treat hematologic malignancies [48]. In total, six CAR-Ts have been approved, four of which are directed against CD19 in B-cell malignancies, primarily diffuse large B-cell lymphoma (DLBCL): axicabtagene ciloleucel (YESCARTA^®^) [49], tisagenlecleucel (KYMRIAH^®^) [50], brexucabtagene autoleucel (TECARTUS^®^) [51]. Lisocabtagene maraleucel (BREYANZI^®^) [52] and two directed against BCMA in multiple myeloma idecabtagene vicleucel (ABECMA^®^) [53] and ciltacabtagene autoleucel (CARVYKTI^®^) [54].

### 4.2. Chimeric Antigen Receptor T-Cell Related Encephalopathy

CAR-T-related encephalopathy (CRES) is a CAR-T-mediated CNS toxicity associated with significant morbidity and mortality that may hinder the expansion of CAR-T therapies. CRES has recently been renamed immune effector cell-associated neurotoxicity syndrome (ICANS) because similar neurotoxic symptoms have been reported with other immune effector cell-engaging therapies, such as bi-specific T-cell engagers (blinatumomab) [55].

ICANS is relatively common, affecting up to 45% of patients with severe, sometimes fatal, neurological symptoms [56,57,58,59]. This syndrome has been observed not only with CARs targeting CD19 but also with those targeting CD22 and BCMA. ICANS can occur with CARs containing either CD28 or 4-1BB costimulatory domains, although the incidence varies: up to 45% in patients with CD28-based CARs compared to only 13% with 4-1BB-based constructs [60,61,62]. The underlying mechanisms of these toxicities are not fully understood, but key features of ICANS include endothelial activation and disruption of the blood–brain barrier, leading to brain edema due to cytokine release by CAR-T cells [58,59,60,63,64,65]. Indeed, a report by Norelli et al. showed that proinflammatory cytokines and myeloid cells are involved in addition to activated T cells [66]. This leakage leads to cerebral edema, hemorrhage, infarction, and necrosis due to intravascular coagulation (DIC) [59]. The latter can be measured in the blood of patients with severe neurotoxicity, as they have significantly elevated DIC markers [60]. Elevated levels of the excitatory NMDA receptor agonists glutamate and quinolinic acid have also been shown in the cerebrospinal fluid of patients with neurotoxicity [67].

Although ICANS can develop without the presence of cytokine release syndrome (CRS), patients typically experience both conditions concurrently (91%). Clinically, CRS resembles septic shock and is characterized by high fever, tachycardia, hypotension, myalgia, respiratory failure, vascular leakage, coagulopathy (disseminated intravascular coagulation, or DIC), and oliguria, which can lead to multiple organ failure in severe cases [45,61,62]. It is a systemic phenomenon that has the potential to affect any organ, including the nervous system. The severity of ICANS is proportional to that of the CRS and is, therefore, closely related to in vivo T-cell expansion, with ICANS occurring when CAR-T levels are highest [59,60]. ICANS typically present 4–5 days after CAR T-cell administration, but delayed onset occurring 3–4 weeks after CAR T-cell treatment has been reported [57]. Clinically, ICANS follows a stereotypical progression, beginning with somnolence, confusion, attention deficit, disorientation, and mild aphasia. These symptoms can then worsen to include aphasia, hallucinations, delirium, myoclonus, and tremors. In severe cases, patients may experience generalized seizures and encephalopathy, leading to coma and potentially death [45,60,66]. EEG findings typically show nonspecific diffuse generalized slowing triphasic waves. Such patients risk developing posterior reversible encephalopathy syndrome (PRES) and acute necrotizing encephalopathy [59,64].

Unlike conventional CRS, for which tocilizumab is routinely used, ICANS does not respond well to IL-6R blockade. Intravenous methylprednisolone and antiepileptic drugs (levetiracetam) appear more effective in this situation [60,64]. Plasmapheresis has been proposed as it is used in thrombotic thrombocytopenic purpura, which shares similar endothelial cell activation mechanisms, but this approach has yet to be validated [68].

ICANS grading and management guidelines have been published by the American Society of Bone Marrow Transplantation (Table 4) [64].

### 4.3. Bispecific T Cell Engager Immunotherapy (BiTEs)

Bi-specific T-cell engagers (BiTEs) can be considered at the interface between ICI and CAR-T, as they are an antibody-based, infused drug, but directly confer a novel T-cell specificity. To date, five BiTEs have been approved, blinatumomab (BLINCYTO^®^), a CD-19-targeting construct indicated as second-line treatment for Philadelphia chromosome-negative relapsed or refractory acute lymphoblastic leukemia [69], tebentafusp (KIMMTRAK^®^), a bispecific gp100 peptide-HLA-directed CD3 T-cell engager indicated for the treatment of HLA-A*02: 01-positive adult patients with unresectable or metastatic uveal melanoma [70], glofitamab, (COLUMVI^®^) a bispecific CD20-CD3 monoclonal antibody used for the treatment of diffuse large B-cell lymphoma [71], mosunetuzumab, (LUNSUMIO^®^), also a CD20-CD3 BiTE is used for the treatment of follicular lymphoma [72], and finally talquetamab, (TALVEY^®^), a humanized GPRC5D-CD3 BiTE is used in the treatment of multiple myeloma [73]. Many more are under development and will reach the market soon [74].

BiTEs induce systemic AEs similar to CAR T cell toxicity with the typical development of fever, headache, neutropenia, and cytokine release syndrome. However, this therapy is also known to induce neurologic adverse events in up to half of treated patients. These include tremors, seizures, confusion, febrile delirium, (leuko)encephalopathy, and cerebellar ataxia. Up to 30% of patients experience Grade 3 symptoms [75,76]. These nAEs typically occur early in treatment (median onset = 9 days) and are generally reversible and manageable with high-dose dexamethasone (3 × 8 mg/day for 4 days) and interruption of the BiTE infusion. Premedication with 20 mg of dexamethasone IV, administered 1 h prior to BiTE infusion, is recommended to minimize the risk of nAE development. Patients experiencing grade 4 symptoms or seizures should be permanently discontinued. Otherwise, treatment may be resumed once symptoms have resolved to grade 1 or have disappeared. Although not fully understood, BiTE nAEs are thought to result from transient neuroinflammation in the CNS due to endothelial adherence of BiTE-activated T cells [75].

## 5. Chemotherapy-Induced Encephalitis

Chemotherapy is known to induce chemotherapy-induced toxic (leuko)encephalopathy, or “chemobrain”, which is clinically characterized by progressive cognitive dysfunction. However, this chronic and progressive form of neurotoxicity is not the only one induced by chemotherapy regimens. In fact, several cytostatic drugs can damage the central nervous system after systemic (intravenous, oral) or topical (i.e., intrathecal, intraventricular, or intraarterial) administration. Cytotoxic drug-associated clinical syndromes are classified into 11 categories: acute encephalopathy, aseptic meningitis, cerebellar dysfunction, cerebral infarction, chronic encephalopathy, cortical blindness, multifocal leukoencephalopathy, posterior reversible (leuko)encephalopathy syndrome (PRES), thrombotic microangiopathy, seizure, and subacute encephalopathy [77]. For the scope of this review, we will only discuss systemic chemotherapy applications and acute complications.

The incidence of CNS toxicity depends on a number of factors, including the chemotherapeutic substance administered, its dosage (single and cumulative), the duration of treatment, the infusion speed, and additional risk factors including preexisting neurological conditions. The cytostatics most associated with CNS toxicity are cytarabine (Ara-C), ifosfamide, and methotrexate (MTX) [77]. These drugs are known to cause acute encephalopathy, which develops within hours to days after infusion. Patients display typical symptoms of somnolence, confusion, disorientation, agitation, and focal stroke-like symptoms such as hemiparesis, ataxia, dysarthria, and possibly even coma [78]. Myoclonic jerks, seizures, and hallucinatory symptoms may occur [79,80]. If untreated, acute encephalopathy may progress to chronic leukoencephalopathy with chronic sequelae.

As above, a broad differential diagnosis including infectious encephalitis, non-convulsive epileptic status, paraneoplastic syndrome, metabolic disorders, and brain metastasis is to be excluded [78]. Cytotoxics commonly associated with acute encephalopathy are 5-fluorouracil (5-FU), cytosine arabinoside (Ara-C), etoposide/VP16 (high dose), ifosfamide (10–15% of patients treated with doses > 1 g/m^2^), interferon-α, interleukin-2, methotrexate (MTX), nitrosoureas (high dose), paclitaxel, procarbazine, and tamoxifen (high dose) [78]. Table 5 provides a non-exhaustive summary of medications, associated symptoms, risk factors, and management.

Cases of acute encephalopathy and focal neurological deficits require urgent intervention to prevent the development of chronic leukoencephalopathy. Once established, there is no treatment available to reverse the chronic changes. Keeping in mind the absence of consensus on the best management of acute toxicity, several treatment approaches have been investigated with variable success rates. Treatment of MTX complications includes CSF drainage, ventriculo-lumbar perfusion with saline, and glucarpidase, a drug catabolizing an enzymatic cleavage degrading MTX into a non-toxic metabolite [81,82]. Unfortunately, leucovorin, routinely given to rescue patients from systemic MTX toxicities, cannot be given intrathecally. One fatal case has been reported [83].

Methylene blue, administered at a dose of 50 mg IV every 4 h, has been used successfully in the treatment of ifosfamide-induced encephalopathy, where it is thought to act as an alternative electron acceptor [84]. Ifosfamide and its metabolites are thought to interfere with thiamine functions. Thus, prophylaxis with 100 mg of thiamine IV every 4–6 h can be empirically administered to prevent ifosfamide-induced encephalopathy [85]. In all cases of acute encephalopathy, discontinuation of chemotherapy is often the only method to prevent further CNS toxicity [77,78,80]. However, none of these approaches have been evaluated prospectively or in a randomized manner.

Subacute encephalopathy is rare but may develop days to weeks after administration of certain cytostatic agents such as cisplatin or MTX, presenting as an abrupt onset of confusion, seizures, and focal signs such as hemiparesis or aphasia. It primarily affects children, but isolated cases have been reported in adults [86]. The mechanism of neurotoxicity is not well understood. Symptoms usually resolve spontaneously, but fatal outcomes have been observed [77].

Cerebellar and brain stem dysfunction with dysarthria, oculomotor disorders, and ataxia are typical complications of cytarabine, usually at cumulative doses over 36 g/m^2^ [77]. Autopsies have revealed widespread Purkinje cell loss. Fortunately, only isolated cases of complication have been reported at lower doses. Risk factors for the development of cerebellar dysfunction include advanced age, renal insufficiency, and elevated alkaline phosphatase. A similar cerebellar syndrome may be seen after treatment with high-dose 5-FU, which is reversible after interruption of therapy, but may recur with re-exposure to the drug [77].

Chemotherapy-induced autoimmune-mediated encephalitis is rare but has recently been described. A case of autoimmune encephalitis with oligoclonal bands in the cerebrospinal fluid has been reported in a 10-year-old patient treated for a pure germinoma in the suprasellar region. Her clinical status improved rapidly with steroids [87]. Similarly, a rare case of anti-Yo autoimmune encephalitis with confusion, behavioral changes, and seizures was reported in a 66-year-old patient receiving a capecitabine/oxaliplatin treatment [88]. Recently, chemotherapy for colorectal cancer has been shown to induce autoantibodies, although the pathophysiology is not yet understood [89].

Posterior reversible encephalopathy syndrome (PRES) typically presents itself with headache, paresis, blurred vision, nausea, seizures, and altered mental status. Risk factors include hypertension, eclampsia/preeclampsia, infection/sepsis, and cancer chemotherapy. Characteristic hyperintense parieto-occipital lesions involving gray and white matter are seen on T2-weighted MRI. These signal abnormalities are transient, and PRES is usually reversible upon withdrawal of the causative agent, but permanent disabilities have been reported. Despite a still largely unknown etiology, PRES is thought to be related to alterations in cerebrovascular autoregulatory control rather than direct drug cytotoxicity. It is hypothesized that exogenous toxins and chemicals may cause direct toxicity to the endothelium of the cerebral blood vessels, resulting in disruption of the blood–brain barrier. Chemotherapeutic drugs for which PRES has been reported include vincristine, oxaliplatin, gemcitabine, and doxorubicin [90,91,92]. No specific therapy has been shown to be effective in PRES; it is usually recommended to control possible hypertension and avoid the chemotherapeutic agent responsible in the future [78].

Targeted therapies may also cause encephalopathies. PRES has been described with several targeted therapies, including tyrosine kinase inhibitors such as sunitinib [93] and sorafenib [94]. Bevacizumab, a monoclonal antibody that inhibits angiogenesis by blocking vascular endothelial growth factor (VEGF), has also been implicated as a cause of PRES, particularly in patients with renal and colorectal cancer [95,96]. Rituximab, a monoclonal antibody targeting CD20 used to treat B-cell malignancies, is also capable of inducing PRES, although the pathophysiologic mechanism remains unknown [97].

Finally, radiation-induced CNS toxicity continues to be a significant cause of morbidity among cancer patients. Although there have been advances in creating more precise and safer radiation techniques, these improvements have been counterbalanced by the growing use of combined radio-chemotherapy regimens, the advent of radiosurgery, and the rising number of long-term survivors. However, these effects tend to develop over the long term and are due to general cerebral toxicity rather than encephalitis [98].

## 6. Conclusions

Although immunotherapy is revolutionizing cancer treatment and its use is rapidly expanding, it is associated with specific and often serious side effects that must be understood by treating physicians. Immunotoxicity and autoimmunity are common complications of checkpoint inhibitors and CAR T cells. Among the wide range of immune-related adverse events, neurological AEs, particularly encephalitis, are rare but represent an adverse outcome if not managed appropriately. The timing of symptom onset is highly variable, making patient monitoring and diagnosis difficult. Patients developing rapidly evolving confusion under ICI treatment should be suspected of having encephalitis. The diagnostic workup should include blood tests, CSF analysis, cerebral MRI, and an EEG. The treatment of choice relies on intravenous corticosteroids. Alternative treatment options include IVIG, rituximab, natalizumab, and plasma exchange therapy. However, current consensus guidelines are based on empirical data. Prospective clinical trials remain an important priority. Targeted therapies and chemotherapy can also induce encephalopathies to a lesser extent, some specific treatments have been discussed, but the main intervention remains treatment discontinuation.

## Figures and Tables

**Table 1:** High and intermediate risk tumor-associated autoantibody-mediated encephalitis.

Selection of High and Intermediate-Risk Tumor-Associated Autoantibody-Mediated Encephalitis	
Autoantibody-Mediated Encephalitis	Related Tumor
**High-risk antibodies (>70% paraneoplastic**)	
Hu	Small and non-small cell lung cancer, neuroendocrine tumors, and neuroblastoma
CV2/CRMP5	Small cell lung cancer and thymoma
Yo	Small and non-small cell lung cancer, breast cancer
Ma2	Testicular cancer, non-small cell lung cancer
SOX1	Small cell lung cancer
PCA2/MAP1B	Small and non-small cell lung cancer, breast cancer
Ri	Small and non-small cell lung cancer, breast cancer
Tr	Hodgkin’s lymphoma
KLHL11	Testicular cancer
**Intermediate-risk antibodies (30–70% paraneoplastic)**	
NMDAR	Ovarian or extraovarian teratomas
AMPAR	Small cell lung cancer, malignant thymoma
GABA A/B	Small cell lung cancer
CASPR2	Malignant thymoma
mGluRs	Hodgkin’s lymphoma

Abbreviations: AMPAR: a-amino-3-hydroxy-S-methyl-4-isoxazolepmpionic acid receptor. CASPR2: contactin-associated protein-like 2; CRMP5: collapsin response mediator protein 5; GABA: gamma-aminobutyric acid; KLHL11: Kelch-like protein 11; MAP1B: microtubule-associated protein 1B; mGluRs: metabotropic glutamate receptor S; NMDAR: N-methyl D-aspartate receptor; PCA: Purkinje cell antibody. Table adapted from [13].

**Table 2:** Approved immune checkpoint inhibitors and indications.

Approved Checkpoint Inhibitors and Their Indications			
Drug Name	Target	First Approval	Indications
Pembrolizumab (Keytruda)	PD-1	2014	Melanoma, NSCLC, HNSCC, HL, PMBCL, UC, microsatellite instable tumors (MSI-H or dMMR) (CRC, endometrial, stomach, small intestine, BTC, RCC, TNBC, uterine cancer)
Nivolumab (OPDIVO)	PD-1	2014	NSCLC, mesothelioma, melanoma, RCC, HL, HNSCC, CRC, UC, esophageal adenocarcinoma, esophageal SCC,
Cemiplimab (LIBTAYO)	PD-1	2018	SCC, NSCLC, Cervical Cancer
Dostarlimab (JEMPERLI)	PD-1	2021	EMA: dMMR endometrial cancer; FDA: dMMR) solid tumors (CRC, endometrial, stomach, small intestine, BTC, RCC, TNBC, uterine cancer)
Atezolizumab (TECENTRIQ)	PD-L1	2016	NSCLC, SCLC, UC, TNBC, melanoma, HCC
Durvalumab (IMFINZI)	PD-L1	2016	NSCLC, SCLC, UC, BTC
Avelumab (BAVENCIO)	PD-L1	2017	Merkel cell carcinoma, UC, RCC
Ipilimumab (YERVOY)	CTLA-4	2011	Melanoma, RCC, CRC, mesothelioma, esophageal SCC
Tremelimumab (IMJUDO)	CTLA-4	2022	NSCLC, HCC
Relatlimab and nivolumab (OPDUALAG)	LAG-3 and PD-1	2022	Melanoma

BTC: biliary tract cancer; CTLA-4: cytotoxic T-lymphocyte-associated antigen-4; CRC: colorectal cancer; dMMR: deficient mismatch repair; HCC: hepatocellular carcinoma; HL: Hodgkin lymphoma; HNSCC: head-and-neck squamous cell carcinoma; LAG-3: lymphocyte activation gene 3; MSI-H: microsatellite instability-high; NSCLC: non-small-cell lung cancer; PD-1: programmed cell death 1; PD-L1: programmed cell death-ligand 1; PMBCL: primary mediastinal large B cell lymphoma; RCC: renal-cell carcinoma; SCC: squamous cell carcinoma; SCLC: small-cell lung cancer; TNBC: triple-negative breast cancer; UC: urothelial cancer.

**Table 3:** Differential diagnosis to exclude and recommended work-up.

Differential Diagnosis and Work-Up	
Differential Diagnosis	Work-Up
Infection (sepsis-induced encephalopathy; bacterial, fungal or viral meningoencephalitis; brain abscess)	Blood tests including culture, CSF analysis including culture and viral PCR (herpes simplex, varicella-zoster), MRI
Brain Metastasis/Leptomeningeal tumor dissemination	CSF analysis, MRI
Metabolic encephalopathy	Blood tests *
Nonconvulsive status epilepticus	EEG
Paraneoplastic limbic encephalitis	Blood tests **, CSF analysis ***, MRI
PRES	MRI
Stroke (ischemia/intracerebral hemorrhage)	MRI
Vasculitis	Blood tests (ANCA), MRI

* Glucose, creatinine, bilirubin, liver enzymes, ammonia, electrolytes, arterial blood gases; additionally, in non-responders to therapy: vitamins B1/12, thyroid function tests, cortisol concentrations, toxicological screening, brain injury biomarkers NfL, S100B, GFAP. ** Antibodies against Anti-HU, antibodies including CASPR2, recoverin, AGNA (Sox1), Titin, Zic4, DNER/Tr, amphiphysin, CV2/CRMP5, Ma1, Ma2/Ta (PNMA2), Ri (ANNA2), TG, TPO, Yo (PCA1), TR, GAD65, NMDAR, GABAR, IgLON5, AMPAR1/2, DPPX, LGI1, glycine receptor, and mGluR5. *** Antibodies against NMDAR, AMPAR1/2, VGKC (anti-LGI1, anti-CASPR2), GlyR, GABAR, etc. Abbreviations: AGNA (SOX1) = anti-glia nuclear antibody; AMPAR1/2 = alpha-amino-3-hydroxy-5-methyl-4-isoxazole propionate receptors 1 and 2; ANCA = antineutrophil cytoplasmic antibody; CASPR2 = contactin-associated protein-like 2; CRMP5 (CV2) = collapsin response mediator protein 5; CSF = cerebrospinal fluid; DPPX = dipeptidyl-peptidase-like protein 6; EEG = electroencephalogram; GABAR = gamma aminobutyric acid B receptor; GAD65 = glutamate decarboxylase 65 kDa isoform; GFAP = glial fibrillary acidic protein; GlyR = glycine receptor; Hu = neuronal nuclear antibody-1; ICI = immune checkpoint inhibitor; LGI1 = leucine-rich glioma-inactivated 1; MRI = magnetic resonance imaging; NFL = light chain neurofilament; NMDAR = N-methyl-D-aspartate receptor; PCR = polymerase chain reaction; PRES = posterior reversible encephalopathy syndrome; Ri (ANNA2) = neuronal nuclear antibody-2; TG = thyroglobulin; TPO = thyroid peroxidase; TR = human thrombin receptor; VGKC = voltage-gated potassium channel; Yo (PCA1) = anti-Purkinje cell cytoplasmic antibody type 1; ZIC4 = zic family member 4.

**Table 4:** Diagnosis and management of ICANS.

Grading and Management ICANS				
**1. Grading of ICANS**				
	**ICANS Grade 1**	**ICANS Grade 2**	**ICANS Grade 3**	**ICANS Grade 4**
Neurological assessment score (by ICE score)	7–9 (mild impairment)	3–6 (moderate impairment)	0–2 (severe impairment)	0 patients in critical condition and/or unable to perform assessment
Depressed level of consciousness (not attributable to other factors (sedation…)	spontaneous awakening	awakens to voice	Awakens only to tactical stimulus	Patient is unarousable or needs vigorous/ repetitive tactical stimulus. Stupor or coma
Raised intracranial pressure	NA	NA	Focal/local edema on neuroimaging	Diffuse cerebral edema on neuroimaging; decerebrate or decorticate posturing or cranial nerve VI palsy; or papilledema or Cushing’s triad
Seizure	NA	NA	Any clinical seizure that resolves rapidly or non-convulsive seizure on EEG that resolves with intervention	Life-threatening generalized seizure (>5 min) or repetitive clinical or electrical seizures without return to baseline in between
Motor findings	NA	NA	NA	Deep focal motor weakness such as hemi or paraparesis
**2. Management recommendations for ICANS**				
**Grade 1**				
IV hydration, vigilant supportive care, aspiration precautions				
Withhold oral intake of food, fluids & medicines, assess swallowing				
Avoid all medications that may cause CNS depression				
Agitated patients may be controlled with low-dose lorazepam (0.25–0.5 mg IV q8h) or haloperidol (0.5 mg IV q6h)				
Search for and follow a possible papilledema				
Cerebral MRI + MRI of the spine if focal peripheral neurological deficits				
Lumbar puncture with measurement of opening pressure				
Daily 30 min EEG until toxicity symptoms resolve, in the absence of seizure prophylactic levetiracetam q12h				
Consider Tocilizumab 8 mg/kg IV or siltuximab 11 mg/kg IV if concomitant CRS				
**Grade 2**				
Consider transferring the patient to ICU if CRES associated with ≥grade 2 ICANS				
Supportive care and neurological work-up as indicated for grade 1 ICANS				
Tocilizumab 8 mg/kg IV or siltuximab 11 mg/kg IV if concurrent CRS				
Dexamethasone 10 mg IV q6h if refractory to anti-IL-6 or for ICANS without CRS				
**Grade 3**				
Transfer to ICU				
Supportive care and neurological work-up as indicated for grade 1 ICANS				
Anti-IL-6 therapy if associated with concurrent CRS				
Dexamethasone 10 mg IV q6h if CRES symptoms worsen despite anti-IL-6 therapy or for ICANS without CRS. Taper once ICANS is resolved to grade 1				
Follow papilledema				
consider repeat MRI every 2–3 days if persistent CRES ≥ grade 3				
**Grade 4**				
ICU monitoring—consider mechanical ventilation for airway protection.				
Supportive care and neurological work-up as indicated for grade 1 ICANS.				
Anti-IL-6 therapy and repeat neuroimaging as described for grades 2 and 3 ICANS.				
High-dose corticosteroids continued until improvement to ICANS grade 1 (methylprednisolone IV 1g/day, followed by a rapid taper: 250 mg q12h for 2 days, 125 mg q12h for 2 days, 60 mgq12h for 2 days).				
Stage ≥ 3 papilledema with CSF opening pressure ≥ 20 mmHg: High dose corticosteroids (methylprednisolone IV 1g/d); elevate the head end of patient’s bed to 30°; hyperventilation to achieve a partial pressure of arterial carbon dioxide (PaCO_2_) of 28–30 mmHg, but maintained for no longer than 24 h; hyperosmolar therapy with mannitol or hypertonic saline; consider neurosurgery consultation for Ommaya reservoir/drain; consider anesthetics for burst-suppression pattern on EEG; metabolic profiling and daily imaging of the brain to prevent rebound cerebral edema, renal failure, electrolyte abnormalities, hypovolemia, and hypotension				

ICE evaluation chart: 1 point for each of the following points: orientation to year, month, city, hospital (4 pts), name 3 objects (3 pts), ability to follow simple commands (show me 2 fingers: 1pt), write a standard sentence (1 pt), count backward from 100 in tens (1pt) iPAP, bilevel positive airway pressure; CNS, central nervous system;; CSF, cerebrospinal fluid; CRES, chimeric antigen receptor-T-related encephalopathy; CRS, cytokine-release syndrome; EEG, electroencephalogram; ICE score, immune effector cell-associated encephalopathy score; ICU, intensive care unit; IV, intravenous; NA, not applicable. Adapted from [25,64].

**Table 5:** Therapy/prophylaxis of chemotherapy-induced CNS toxicity. Adapted from [78].

Acute Chemotherapy-Induced CNS Toxicity and Management					
Drug	Symptoms	Frequency	Threshold Dosage	Risk Factors	Therapy/ Prophylaxis
Methotrexate (MTX)	Acute reversible encephalopathy	Rare	>0.5 g/m^2^	Whole-brain radiotherapy	Frequently self-limiting
	Subacute encephalopathy	Very rare	After 2nd or 3rd iv application	Increased levels of Homocysteine	Dextromethorphan
	Chronic encephalopathy	Infrequent after MTX alone	>0.5 g/m^2^	Whole-brain radiotherapy, intrathecal MTX	None
Ara-C	Cerebellar dysfunction plus possible acute encephalopathy	Frequent, if dose > 36 g/m^2^	cumulative dose >36 g/m^2^	Renal insufficiency, increased alkaline phosphatase, age > 60, neurological comorbidity	Frequently self-limiting
5-Fluorouracil	Acute encephalopathy	Rare		Low dihydropyridine dehydrogenase	None
	Cerebellar dysfunction plus facultative additional CNS symptoms	Rare		Allopurinol increase, N-Phosphonoacetyl- aspartate (PALA) thymidine	Often self-limiting
	Inflammatory multifocal leukoencephalopathy	Rare		Levamisole	None
Ifosfamide	Acute encephalopathy	Up to 30%		High dose, renal and hepatic insufficiency, low albumin	Frequently self-limiting. methylene-blue, thiamine
